# Music therapy and anxiety: A bibliometric review from 1993 to 2023

**DOI:** 10.1097/MD.0000000000037459

**Published:** 2024-03-29

**Authors:** Tingting Lun, Yuecai Chen, Jingcai Liu, Li Li, Jin Yu, Meng Xiang

**Affiliations:** a Clinical School of Acupuncture, Moxibustion and Rehabilitation, Guangzhou University of Chinese Medicine, Guangzhou, China; b Guangzhou International Economics College, Guangzhou, China; c College of TCM health care, Guangdong Food and Drug Vocational College, Guangzhou, China.

**Keywords:** anxiety, bibliometrics, CiteSpace, music therapy, VOSviewer

## Abstract

**Background:**

Music therapy (MT) has received increasing attention from scholars in the efficacy treatment of anxiety symptoms, which is of great significance to human physical and mental health. The visual mapping functionality of CiteSpace and Vosviewer software was applied in this study to assess the status of MT in the treatment of anxiety symptoms.

**Methods:**

In order to find research on MT and anxiety that were relevant for this research, we searched the Web of Science database. We also utilized CiteSpace and VOSviewer software to examine institutions, journals, authors, publications, and keywords for scientometric and visual analysis.

**Results:**

Our findings show that since 2009, the field has developed rapidly and publications on MT and anxiety have gradually increased. The journal Complement Therapies In Medicine published the most relevant articles, the Cochrane Database Of Systematic Reviews journal had the highest citation frequency, and the United States had the most publications. The majority of the top academic institutions in the region are found in the United States, with the University of London having the most publications. The evolution of this field was significantly influenced by Gold C., the author with the most publications, and Bradt J., the author with the most co-citations. The topics of anxiety, nursing, cancer, and pain management have been the focus of this research.

**Conclusion:**

This study has the potential to increase public understanding of MT and anxiety as well as mental health awareness, all of which are crucial for lowering the prevalence of mental diseases.

## 1. Introduction

Statistics show that the prevalence of anxiety and depressive illnesses has dramatically increased by over 28% globally, with women and young people being particularly impacted.^[1]^ The system for providing mental health services has faced multiple challenges in recent years as a result of the increasing number of complaints of anxiety disorders.^[2,3]^ Both pathological and temporary anxiety experience will have negative affect on their physical and mental health in daily life,^[4]^ such as nervousness, anxiety, fear, irritability and other symptoms. Meanwhile, the experience of anxiety may lead to long-term mental tension and accelerate the blood circulation of the whole body, which may increase the blood supply burden of the heart and increase the risk of cardiovascular diseases.^[5]^ It may lead to unnormal and inefficiency work, even decreased body immunity, insomnia and other problems, and gradually worsen the cycle. At present, there are many complementary and alternative therapies used to intervene anxiety emotional state, among which music therapy (MT) is further promoted and used as a simple, convenient and effective method.

MT refers to the use of music to alleviate physical and psychological ailments and discomforts. It is able to change our emotional state through the auditory perception of music and participation in music. The effects of MT can increase our excitement and relaxation while lowering heart rate, blood pressure, and respiratory rate.^[6]^ In addition to changing the indicators of cardiovascular circulation, modern research has found that music has certain positive effects on the endocrine system, nervous system^[7]^ and so on. And these changes can play a certain objective role in whether music relieves anxiety. However, MT is an interdisciplinary field, including mathematics, natural science, behavioral science, social science and art and so on, which cannot be distinguished uniformly by reductionism.^[8]^ Therefore, a holistic study is needed to summarize relevant rules.

The amount of research on MT for anxiety symptoms has gradually expanded in recent years. The development pattern and research hotspots in the field of MT for anxiety can be further exposed using scientometrics and visualization analysis with the assistance of well-known scientific knowledge map analysis tools.^[9]^ The scientometrics and data visualization of the context were generated by the citation visualization software programs CiteSpace^[10]^ and VOSviewer.^[11]^ They approach this by analyzing citations in local literature, presenting the pattern, structure, and distribution of scientific information through visualization, and creating a scientific knowledge map out of the visualization graphs that result from the research.^[12]^ In order to investigate current research questions and directions, to highlight the growth patterns and research frontiers in this area, and to give resources for future growth. we use these software tools in this study to analyze the development status and trends of research on MT for anxiety.^[13]^ Therefore, in this study, we use these tools to analyze the development status and trends of research on MT for anxiety, in order to explore hot research problems and directions, reveal the development dynamics and research frontiers in this field, and finally provide references for its further development.^[13]^

## 2. Collecting data and research methods

### 2.1. Collecting data

**Figure 1.**
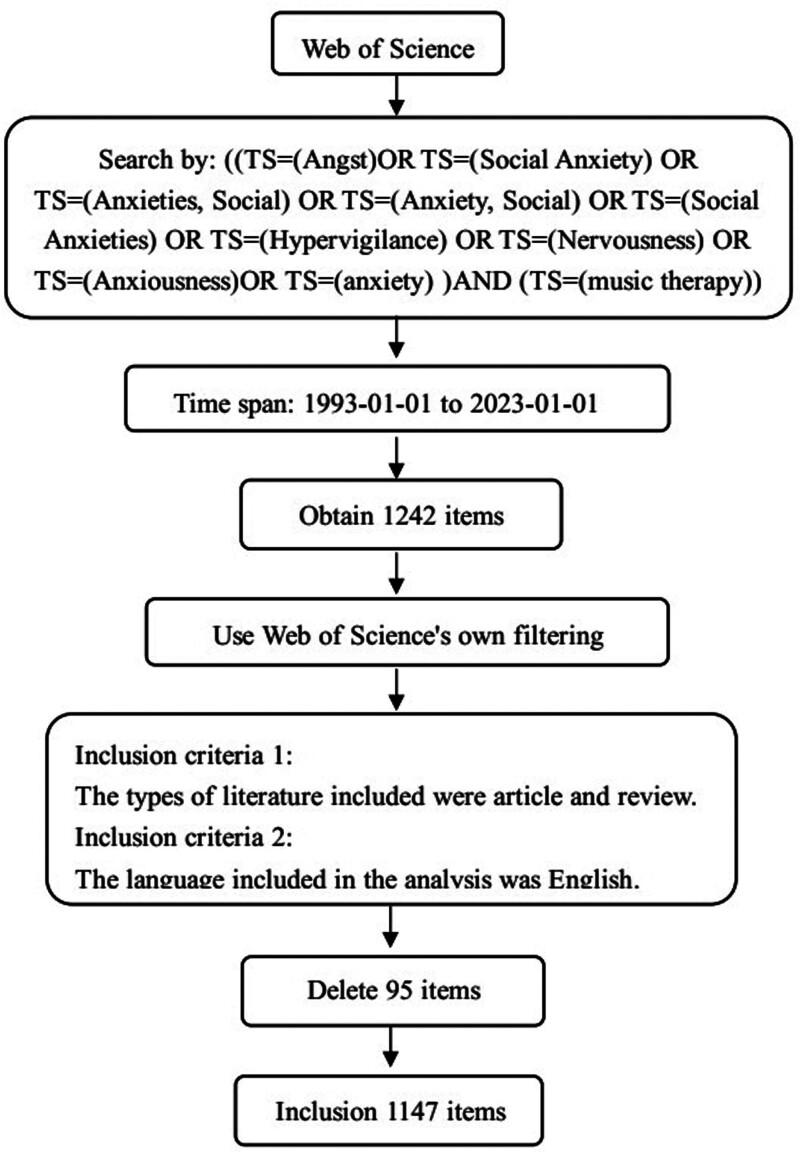
Flowchart of the literature collection.

The Web of Science Core Collection (WOSCC) database, which contains information from 1 January 1993 to 1 January 2023, provided the data for this study. The search strategy was using topic (TS) in the form [(TS = (Angst) or TS = (Social Anxiety) or TS = (Anxieties, Social) or TS = (Anxiety, Social) or TS = (Social Anxieties) or TS = (Hypervigilance) or TS = (Nervousness) or TS = (Anxiousness) or TS = (anxiety)] and TS = (music therapy). 1242 articles were discovered, and then the items were refined according to the literature type using the filter provided by WOS, only the article and review items were retained. Therefore, 70 items were excluded and all included documents for analysis were in the English language, thus excluding 25 items. Finally, this study included 1147 items (Fig. 1). The entire literature was searched and screened on the same day. These retrieved research reports were imported into CiteSpace software in plain text format with complete records and cited references; no duplicate records were discovered.

### 2.2. Research methods

In order to generate annually and cumulative annual publication graphs for this study’s analysis of the output and trends of relevant publications globally, Microsoft Excel 2019 was used. CiteSpace 6.2.R2 and VOSviewer 1.6.17 were used to create scientific knowledge map in the field of MT and anxiety research. Both software programs are Java-based information visualization tools.

The collaborative networks (including countries, institutions and authors) and co-occurrence networks (strongest burst keywords, citations) were produced by CiteSpace. A larger node in the CiteSpace network mapping denotes a greater quantity of publications or a higher co-occurrence frequency. Through quantitative analysis and view visualization, CiteSpace can show research trends in particular domains, assisting in revealing the growth, hotspots, and frontiers of scientific inquiry.^[10]^

Cluster views and density views are produced using VOSviewer, respectively. The cluster to which a node belongs shows the intensity of co-occurrence (or co-citation) and co-occurrence (or co-citation) of the relationship between nodes through its color in the cluster view, its size and the frequency of co-occurrence.^[11]^ In the density view, a color is assigned to each plot node based on how dense the objects nearby are. The closer it is near dark blue, the higher the density. The density increases with proximity to dark blue. On the other hand, density decreases as it gets closer to yellow.

## 3. Results

### 3.1. Publication analysis

**Figure 2.**
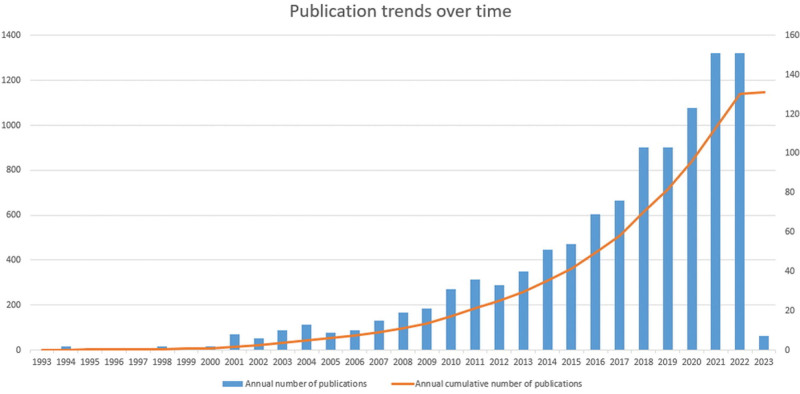
Trend chart of the number of publications.

After searching and filtering, 1147 publications between January 01, 1993 and January 01, 2023 were found in WOS database. A generally positive upward trend can be seen in the progressive rise in publications in the area of MT and anxiety research. Before 2008, there were only a few publications, but after 2009, the number of publications increased rapidly (Fig. 2), probably due to the late start of clinical practice in the field of MT and the lack of attention to mental health at that time.

### 3.2. Country/region analysis

**Figure 3.**
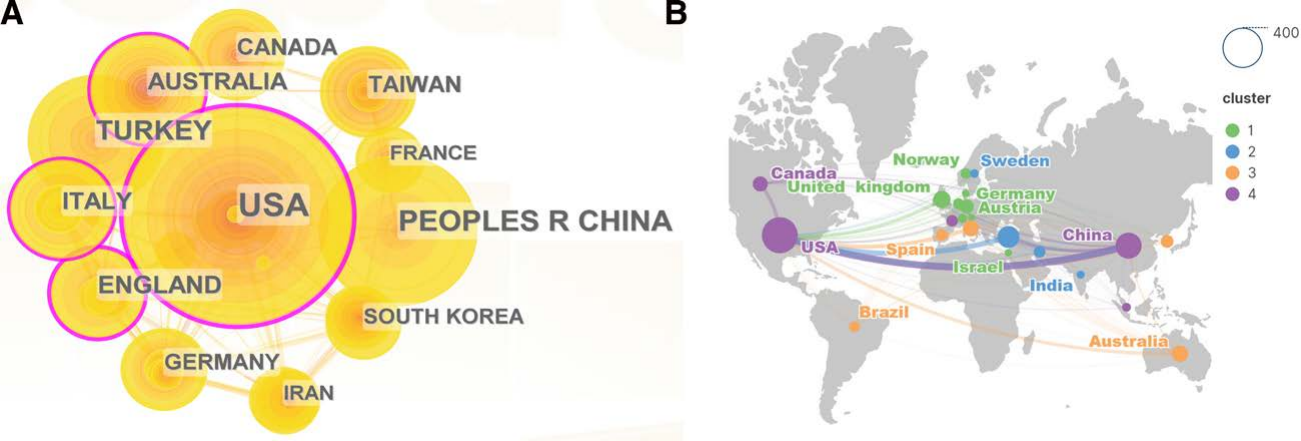
A. An analytical graph of the countries cooperation network. B. The distribution of countries.

The scientific research differences and distribution of MT and anxiety research in each country can be better described by the regional distribution map of countries. Analysis of the national collaboration network through CiteSpace (Fig. 3A). 77 different nations or regions have contributed to publications on MT and anxiety. Among them, in terms of publications, the United States published the most – 297 articles, or 25.89% of the total – and was the most active in the field of MT and anxiety research. With 207 articles, or 18.05% of the total, China came in second. Third place with 108 articles is Turkey, followed by the England with 67 articles, Australia with 63 articles, Italy with 57 articles, Canada with 53 articles, Germany with 44 articles, South Korea with 38 articles, and France with 31 articles (Table 1).

**Table 1:** Top 10 countries ranked by centrality and count.

Rank	Country/region	Centrality	Count	Percentage (%)
1	United States	0.43	297	25.89
2	China	0.09	207	18.05
3	Turkey	0.01	108	9.42
4	England	0.24	67	5.84
5	Australia	0.15	63	5.49
6	Italy	0.16	57	4.97
7	Canada	0.09	53	4.62
8	Germany	0.06	44	3.84
9	South Korea	0	38	3.31
10	France	0.07	31	2.70

The purple circle denotes centrality, with the study’s core constituting its most crucial component. A node is deemed essential if its centrality is more than 0.1, which is represented by the purple circle. The nations with a centrality larger than 0.1 that have a considerable impact on international scientific research cooperation are the United States, England, Italy, and Australia. The worldwide map displays the nations or regions that have made contributions in this field (Fig. 3B).

### 3.3. Institutional cooperation networks analysis

**Figure 4.**
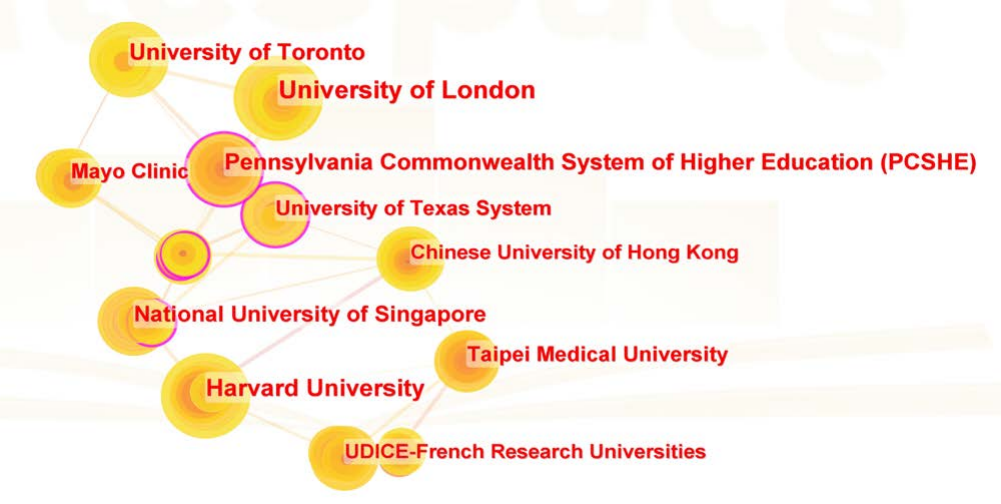
Graph of the institutions cooperation network.

The development of active cooperation networks between universities can explore more prospects for academic interaction and learning as well as support the ongoing advancement of connected research.^[14]^ Figure 4 illustrates the inter-institutional collaboration in the field of MT and anxiety research. Figure 4 and Table 2 indicate that the institutions that published more articles were mainly universities, with the University of London publishing 24 articles and Harvard University 21 articles. The University of Toronto, Pennsylvania Commonwealth System of Higher Education (PCSHE), National University of Toronto Singapore, Taipei Medical University, University of Texas System, Mayo Clinic, UDICE-French Research Universities, Chinese University of Hong Kong, University of Melbourne, and other significant research institutes.

**Table 2:** Top 10 institutions ranked by centrality and count.

Rank	Country	Centrality	Count	Institutions
1	England	0.05	24	University of London
2	United States	0.03	21	Harvard University
2	Canada	0.04	18	University of Toronto
3	United States	0.13	17	Pennsylvania Commonwealth System of Higher Education (PCSHE)
3	Singapore	0	15	National University of Singapore
4	China	0.07	14	Taipei Medical University
5	United States	0.14	13	University of Texas System
5	United States	0	13	Mayo Clinic
5	France	0.02	12	UDICE-French Research Universities
5	China	0	12	Chinese University of Hong Kong
5	Australia	0.1	11	University of Melbourne

### 3.4. Network analysis of journals and co-cited journals

**Figure 5.**
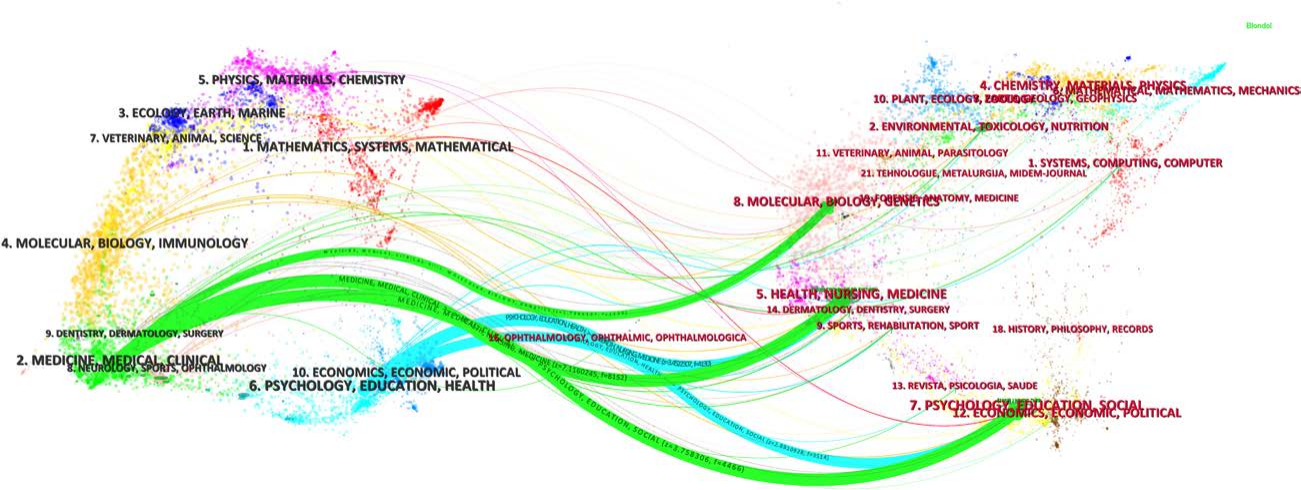
The dual-map overlay of journals with publications.

Articles on MT and anxiety are published in 482 journals. The journal distribution of the cited literature is shown on the left side of the dual journal mapping graphic (Fig. 5), which represents the primary fields and subjects used in science mapping. The key cited fields and research foundation of science mapping are represented on the right side by the journal distribution of the relevant referenced literature. Link trajectories offer an interdisciplinary understanding of the field, curves are citation linkages, z-score functions show more fluent trajectories, and coarser links suggest higher scores.^[14]^ In the circumstances, publications in the fields of clinical medicine, dental surgery, neurology, sports, surgery (green trajectory) are clearly influenced by molecular, biology, genetics (*z* = 1.73, *f* = 2236), nursing, health, surgery, dermatology, sports, rehabilitation, philosophy, history (*z* = 7.12, *f* = 8152) and psychology, education, economics, sociology, and political science (*z* = 3.76, *f* = 4466). Additionally, publications in the fields of psychology, education, economics, health, politics (blue trace) are influenced by publications in the fields of nursing, medicine, health (*z* = 3.45, *f* = 4130) and psychology, education, society (*z* = 2.89, *f* = 3514).

The top 10 journals by number of articles were determined by organizing and ranking the contained material according to the quantity of articles published (Table 3). The majority of the study literature on MT and anxiety is found in publications for nursing, psychology, and systematic reviews. 263 articles, or 21.7% of the total number of articles, were published by the top 10 journals by the number of articles. Classified by the number of publications, *Complement Therapies in Medicine* ranked first with 37 articles. The second and third ranked were J*ournal of Clinical Nursing* and *Cochrane Database of Systematic Reviews, Complementary Therapies in Clinical Practice*, with 35 and 29 articles, respectively. Overall, the United States has 5 of the top 10 journals in terms of the quantity of articles published, with England coming in second with 3 of the top 10 journals.

**Table 3:** Top 10 academic journals based on publications.

Rank	Periodicals	Publications	Citations	Country	Category zone	IF (2022)	CiteScore
1	Complement Therapies in Medicine	37	742	United States	Q2	3.335	3
2	Journal of Clinical Nursing	35	1115	England	Q1	12.008	7.6
3	Cochrane Database of Systematic Reviews	29	2032	England	Q1	4.423	5.3
3	Complementary Therapies in Clinical Practice	29	359	Netherlands	Q2	3.398	1.94
4	Supportive Care in Cancer	24	536	United States	Q2	3.191	2.55
5	Holistic Nursing Practice	24	207	United States	Q4	1.165	0.62
6	Plos One	19	431	United States	Q2	3.752	5.6
7	Journal of PeriAnesthesia Nursing	18	191	United States	Q4	1.230	0.44
8	Psychiatria Danubina	17	26	Croatia	Q4	2.561	0.9
9	Journal of Advanced Nursing	17	481	England	Q1	3.057	4.3

**Figure 6.**
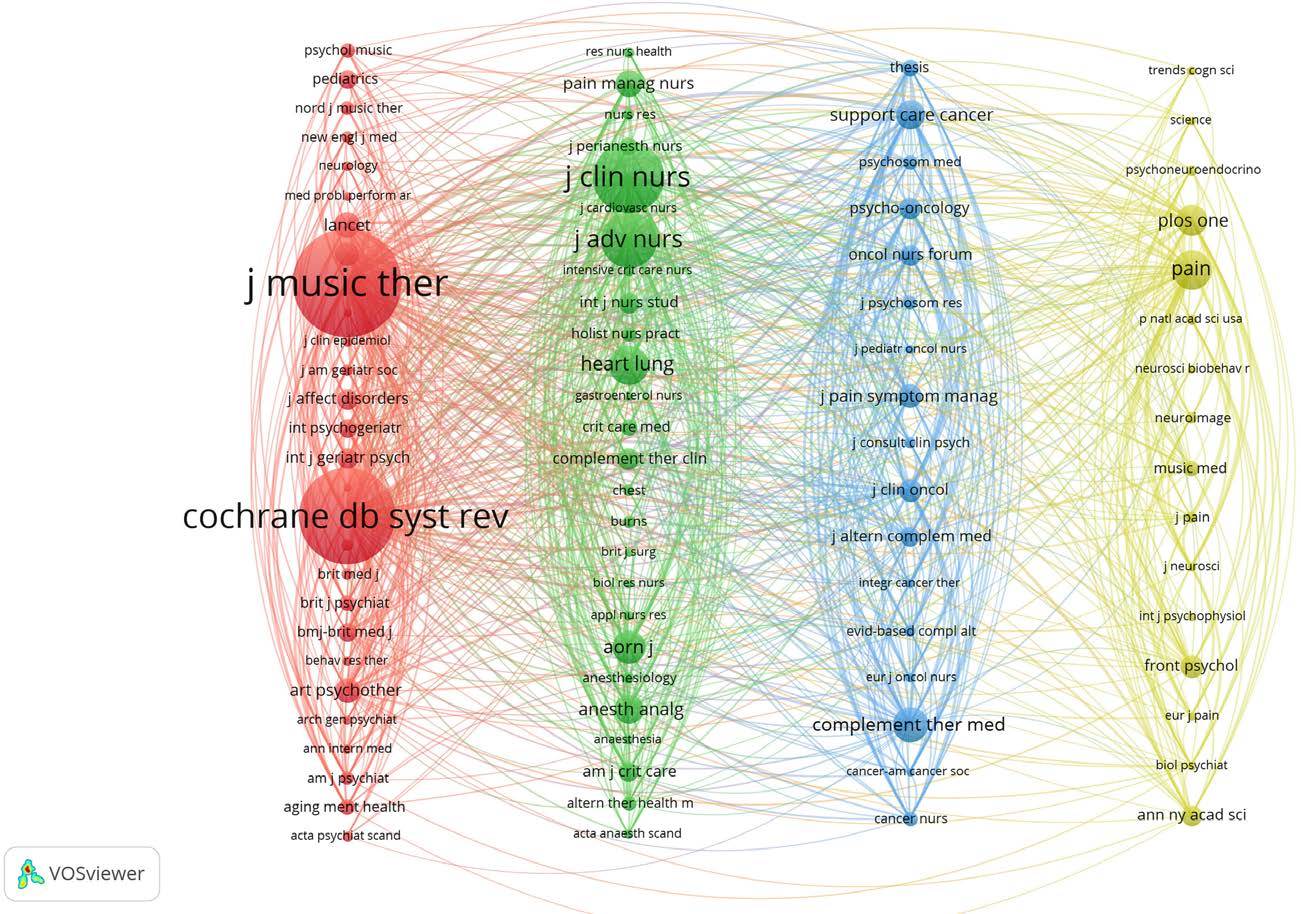
Graph of co-cited journals analysis.

We utilized Pajek software^[15]^ to arrange journals into columns based on various clusters, with nodes signifying journals, and 87 journals will be subjected to citation co-citation analysis using the VOSviewer program, with a 90-citation threshold. The number of times the journal is cited is indicated by the size of the node area, and the intensity of the association is shown by the lines connecting the nodes. Four distinct color clusters were discovered (Fig. 6). The red cluster is mainly journals in the field of MT and psychiatry, focusing on the application and standard establishment in psychiatric disorders and MT; the blue and green clusters are mainly nursing and oncology journals, which tend to conduct more evidence-based research on psychology, oncology, anesthesiology, and complementary and alternative medicine, and are committed to promoting the application of MT. The yellow cluster is mainly a comprehensive journal of neuropsychology, which contains pain, cognition and other research, and provides information on MT in pain management and neuropsychology. In general, the research fields of MT and anxiety show strong interdisciplinary characteristics.

Table 4 and Figure 6 show that the most co-cited is *Journal of Music Therapy* (1164), This was followed by *Cochrane Database of Systematic Reviews* (1029), *Journal of Clinical Nursing* (759), and *Journal of Advanced Nursing* (601) and *Pain* (420).

**Table 4:** Top 5 co-cited journals ranked by centrality and counts.

Rank	Co-cited count	Journal	Centrality	Journal
1	1164	Journal of Music Therapy	0.08	Journal of Advanced Nursing
2	1029	Cochrane Database of Systematic Reviews	0.07	Journal of Music Therapy
3	759	Journal of Clinical Nursing	0.07	Frontiers in Psychology
4	601	Journal of Advanced Nursing	0.07	Journal of Affective Disorders
5	420	Pain	0.06	Aorn Journal

### 3.5. Author and co-cited author network analysis

**Figure 7.**
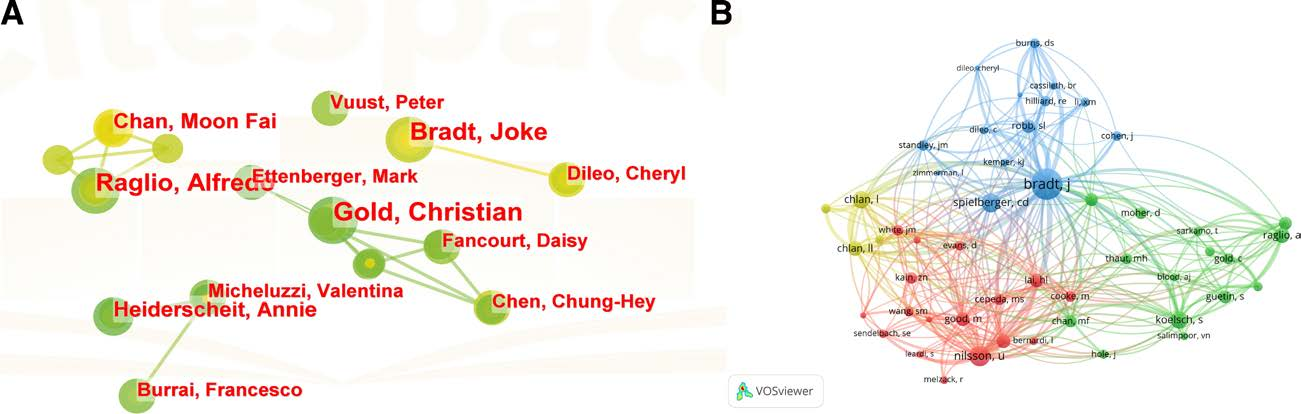
A. Graph of the author collaboration network. B. Graph of the co-cited author collaboration network.

The CiteSpace produced the author cooperation network graph (Fig. 7A), which included a total of 4283 authors who contributed to 1147 articles. The size of a node indicates its number of publications, the top 5 authors by number of publications are shown in Table 5, with Gold, Christian coming in first (8 publications), Raglio, Aifredo coming in second (7 publications), and Bradt Joke coming in third (7 publications).

**Table 5:** Top 5 co-cited authors and active authors.

Rank	Co-cited author	Cited times	Co-cited author	Centrality	Author	Count
1	Bradt J	410	Chlan LL	0.1	Gold C	8
2	Nilsson U	217	Cepeda MS	0.09	Raglio A	7
3	Spielberger CD	185	Spielberger CD	0.09	Bradt J	7
4	Koelsch S	170	CHLAN L	0.07	Chan MF	6
5	Chlan L	165	Hanser SB	0.07	Heiderscheit A	5

Figure 7B displays the network of co-cited authors for all the co-cited authors, Bradt J of Drexel University’s School of Nursing and Health Professions was first with 410 citations, Next were Nilsson U and Spielberger CD with 217 and 185 citations, respectively, and the fourth and fifth co-cited authors were Koelsch S (170 citations) and Chlan L (165 citations). Using the calculating centrality among all co-cited authors, we find that Chlan LL in author collaboration is the author with higher centrality (0.1) over the past 30 years, and other authors have lower centrality, which indicates that the collaboration among authors is more scattered and should be strengthened in the future.

### 3.6. Keyword analysis

#### 3.6.1. Co-occurrence analysis of keywords.

**Figure 8.**
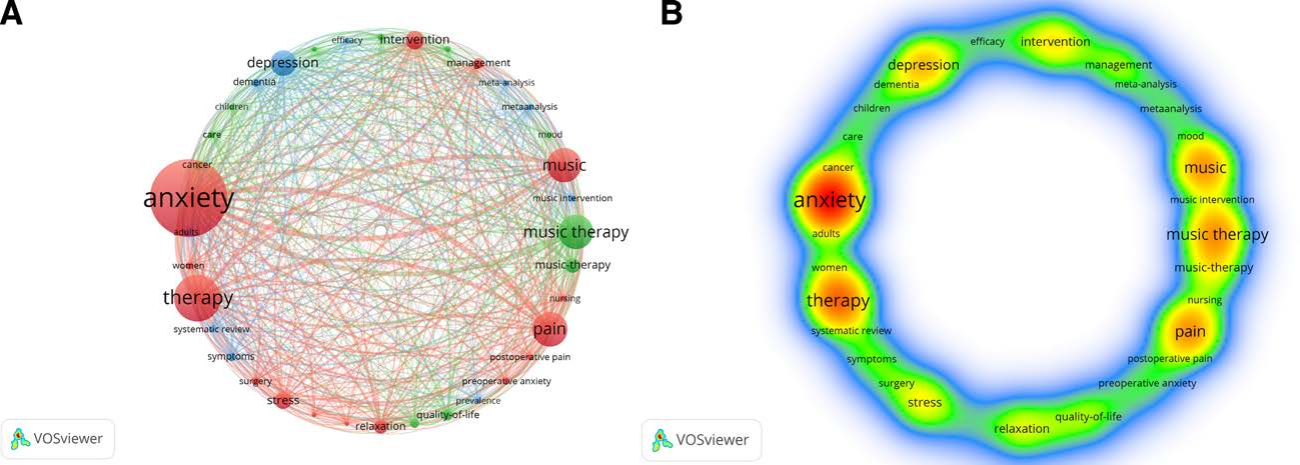
A. Map of co-occurring keywords with occurrences. B. Map of keywords density.

The research hotspots, core topics and values in this field of MT and anxiety research are displayed on the keyword co-occurrence map. Keywords are concise summaries of the article’s topic. In the field of MT and anxiety, 3424 keywords were present as of January 2023. MT and anxiety-related terms were visually analyzed using Pajek and VOSviewer software, with the frequency of co-occurrence of the display set to at least 42. As a result, 36 important keywords in all were included (Fig. 8A). There were 3 different color clusters; the red cluster was primarily concentrated in the domains of anxiety, pain, and MT, while the green cluster was primarily concentrated in the fields of MT, quality of life, and cancer. According to Table 6, the blue clusters are primarily connected with adulthood, depression, and dementia. Table 7 lists the combination of high-frequency keywords with a co-occurrence frequency of more than 100 in order to help researchers better grasp the particular condition of keywords. As displayed in Figure 8B and Table 7, the representative keywords and trending subjects in the field include “MT” (415 times), “anxiety” (414 times), “therapy” (404 times), “pain” (199 times), and “intervention” (157 times).

**Table 6:**
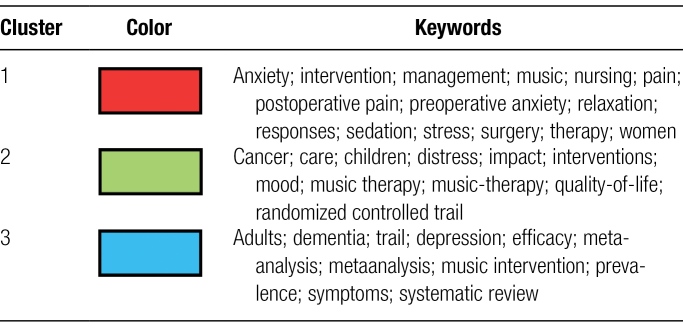
Clusters of co-occurring keywords in areas.

**Table 7:** Top 10 keywords.

Rank	Keyword	Occurrences	Rank	Keyword	Occurrences
1	music therapy	415	6	depression	138
2	anxiety	414	7	relaxation	115
3	therapy	404	8	quality of life	114
4	pain	199	9	randomized controlled trial	105
5	intervention	157	10	stress	103

#### 3.6.2. Keywords with the strongest citation bursts.

Burst keywords refer to keywords that are frequently cited throughout time and may represent cutting-edge topics in this research field. The top 15 keywords are listed in Table 8. Light blue bars indicate that the reference hasn’t yet begun to appear, whereas dark blue bars indicate that it has. Red lines show the burst time. The most cited and trendy keywords included relaxation, care, and preoperative anxiety in the early stage, indicating that relaxation, care, and preoperative anxiety were hot topics for early research in this field. Vital signs (2017–2019), heart rate (2014–2018), and mental health (2017–2020) are recent popular keywords. Three research trends are as follows:

**Table 8:**
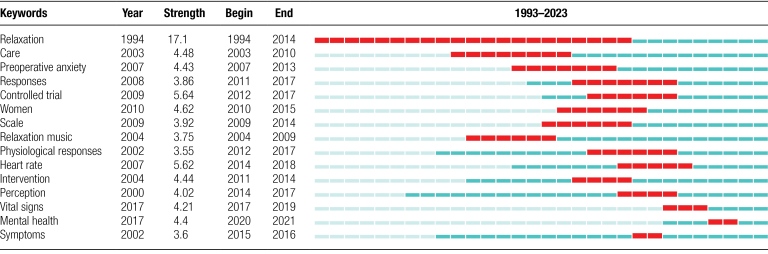
Top 15 keywords with the strongest citation bursts.

1. mental health: A person’s health is multi-dimensional, including physical, mental and social health nowadays.^[16]^ With the change of social environment, the problem of mental disorders has become more and more prominent. Currently, there are some side effects associated with pharmacological therapy for mental diseases. The moderating impact of non-drug therapy on mental psychology attracts more and more attention.^[17]^ It is vital to encourage the development of a mental health service system in this situation.
2. vital signs: The data of objective vital signs serve as the foundation for the clinical evidence-based MT research, which can help to advance the field of MT. Pulse rates,^[18]^ electroencephalograms,^[19]^ magnetic resonance imaging,^[19]^ and other vital signs have recently become important for the study of MT and the monitoring of anxiety symptoms. These vital indicators serve as a particular point of reference for the investigation of the mechanism of action of MT.
3. heart rate: Music can powerfully evoke and regulate emotions and emotions, as well as changes in heart activity, blood pressure and respiration.^[20]^ Heart rate can be detected easily and inexpensively, and there are several fundamental studies on the changes of the body that correspond to the heart rate condition in the early stage,^[21]^ so this may be one of the reasons why it has become a burst keyword, and future research can refer to this index.

Although there are some burst keywords, it is seen that there won’t be any hot keywords that are important after 2020. This may be the reason of MT covers a wide range of areas, and the impact of the pandemic means that many MT are unable to be carried out well, thus no hot research direction has been established.

### 3.7. Citation analysis

#### 3.7.1. Co-citation analysis.

**Figure 9.**
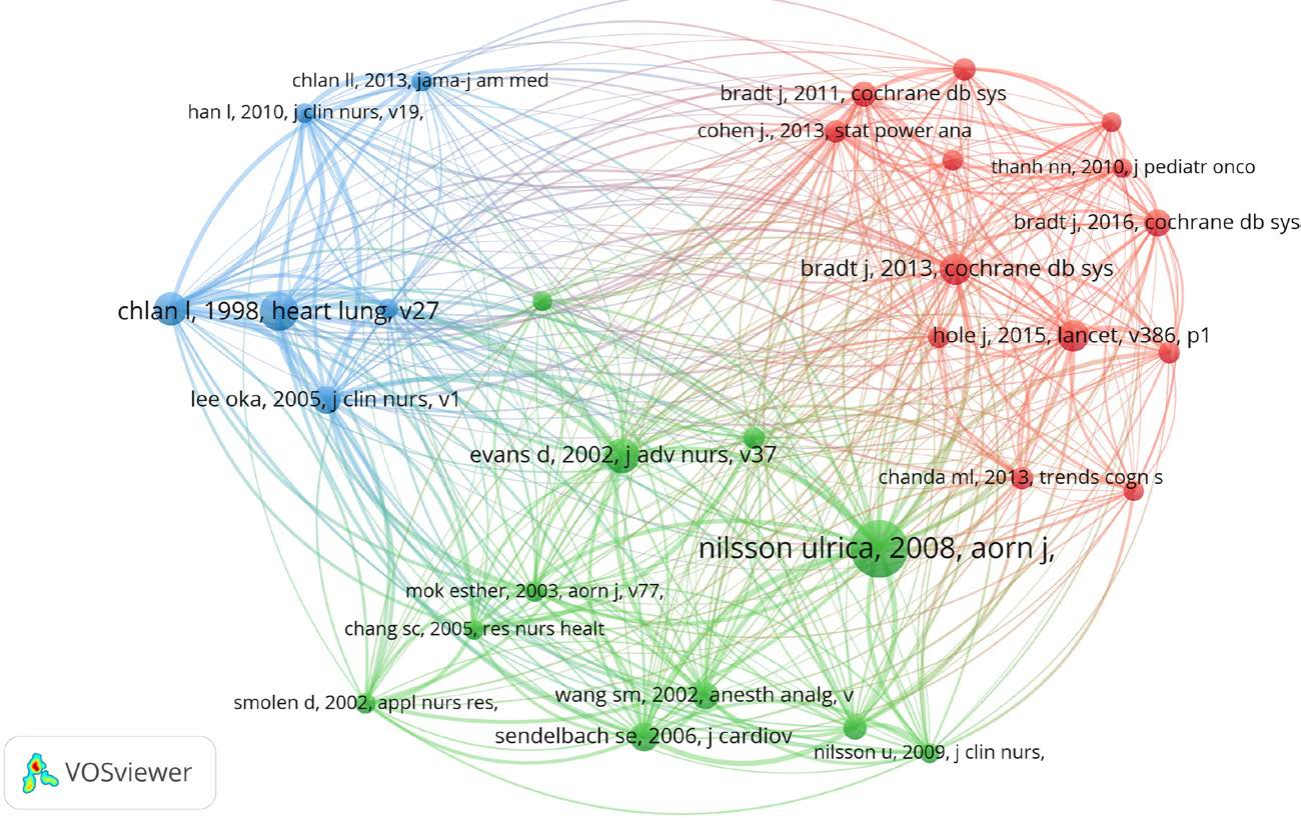
Map of co-cited references with citations.

Highly cited research is a valuable source of scientific knowledge in any research field. It is also used to assess the level of scientific research quality and the influence status of various nations, institutions, and individuals.^[12]^ Highly cited research also reveals the level of the field’s research and the direction in which it is developing, providing the rationale for identifying research frontiers and hotspots. Utilizing VOSviewer, we drew a total of 34,231 references to analyze the co-citation of the literature. The referenced literature was then analyzed for co-citations in thirty research, and a network was generated in the field of MT and anxiety (Fig. 9). Table 9 lists the top 10 most frequently cited articles, including 5 reviews and 5 articles, The most frequently cited article is “The anxiety and pain reducing effects of music interventions: a systematic review “, this article has 117 citations and is written by Nilsson U. Among them, author Bradt J. has 3 reviews in the top 10 co-cited literatures, all of which are published in the *Cochrane Database of Systematic Reviews*.

**Table 9:** Top 10 co-cited references based on citations.

Rank	Title	First author	Periodicals	Country	Citations	Year	Document type
1	The anxiety- and pain-reducing effects of music interventions: a systematic review	Nilsson U	Aorn Journal	United States	117	2008	Review
2	Effectiveness of a music therapy intervention on relaxation and anxiety for patients receiving ventilatory assistance	Chlan L	Heart Lung	United States	79	1998	Article
3	The effectiveness of music as an intervention for hospital patients: a systematic review	Evans D	Journal of Advanced Nursing	England	71	2002	Review
4	Effects of music therapy on anxiety in ventilator-dependent patients	Wong HLC	Heart Lung	United States	68	2001	Article
5	Music interventions for preoperative anxiety	Bradt J	Cochrane Database of Systematic Reviews	England	65	2013	Review
6	Music as an aid for postoperative recovery in adults: a systematic review and meta-analysis	Hole J	Lancet	England	64	2015	Review
7	Effects of music therapy on physiological and psychological outcomes for patients undergoing cardiac surgery	Sendelbach SE	Journal of Cardiovascular Nursing	United States	59	2006	Article
8	Music interventions for improving psychological and physical outcomes in cancer patients	Bradt J	Cochrane Database of Systematic Reviews	England	59	2016	Review
9	Music and preoperative anxiety: a randomized, controlled study	Wang SM	Anesthesia and Analgesia	United States	58	2002	Article
10	Music interventions for improving psychological and physical outcomes in cancer patients	Bradt J	Cochrane Database of Systematic Reviews	England	56	2011	Review

#### 3.7.2. Co-cited references with the strongest citation bursts.

Table 10 shows the top 15 references that were most frequently mentioned from 1993 to 2023. Almost all of them started to gain popularity after 2009, showing that there is still space for more investigation and advancement in the field of MT and anxiety research. Nilsson U, Bradt J, and Kuhlmann AYR are the top 3 authors with the strongest burst sources, and the article published by Nilsson U’s group in Aorn Journal had the highest citation intensity (n = 14.03 citation bursts). They summarized the anxiety and pain reduction effects of music interventions.^[22]^ Prior research by their team included randomized clinical trials on the effects of music treatments on oxytocin levels during post-cardiac surgery bed rest^[28]^ and on stress responses following heart surgery.^[29]^

**Table 10:**
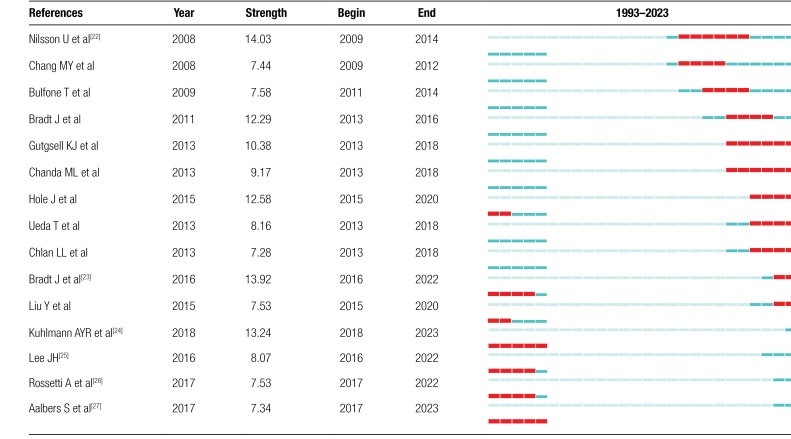
Top 15 co-citation references with the strongest citation bursts.

Bradt et al^[23]^reviewed that music interventions improve psychological and physical outcomes in cancer patients. Because living with cancer can cause a wide range of emotional, physical and social distress. Cancer patients’ symptoms and adverse effects from treatment can be reduced by the use of music treatments, and MT may be an effective and affordable way of coping for cancer patients.^[30]^ Their team also studied MT in the fields of preoperative anxiety,^[31]^ dental care anxiety,^[32]^ reducing the stress and anxiety of patients with coronary heart disease^[33]^ and anxiety of patients with mechanical ventilation,^[34]^ mainly using the method of review. Kuhlmann et al^[24]^ conducted a meta-analysis of MT in the treatment of surgical anxiety and pain, and excavated the bidirectional relationship between physical and psychological emotions.

There are 2 references whose reference burst will end in 2023 and 3 articles whose reference burst will end in 2022, and these articles have been extensively recently, showing that the concepts in these references are hot topics that frequently get attention. As previously indicated, Bradt J and Kuhlmann AYR studied the systematic review of MT and cancer, pain management and anxiety. Aalbers et al^[27]^studied the study of MT in depression and anxiety. Rossetti et al^[26]^studied the anxiety of cancer patients after chemotherapy, and Lee^[25]^ studied the intervention of music on pain. According to studies, depression and anxiety are related to how seriously people perceive their pain, and persistent acute pain will result in more emotional health problems.^[35]^ Therefore, we need to pay more attention to the study of physical and mental changes caused by MT intervention, so that MT can be used as one of the links for the transformation of physical and mental states with the help of objective methods.

## 4. Significance

### 4.1. Significance of published content to research

MT belongs to a kind of noninvasive and emerging complementary and alternative therapy, which integrates music, medicine and psychology as an inter-discipline. This study found that it has been applied and explored in the field of special children, adult mental health, rehabilitation, spiritual field, elderly field, analgesia, maternity field and so on. However, due to many complex variables in culture, social values, technology, artistic functions, spiritual beliefs, disease concepts and human physical and mental knowledge, the whole historical development process has been relatively scattered and lacks a large number of systematic and authoritative studies. Studies have shown that music can affect the autonomic nervous system through the hypothalamic-pituitary-adrenal axis by stimulating specific areas of the brain, and then regulate the physiological functions of the immune system, cardiovascular system, respiratory system and digestive system.^[36]^ In the future, the mechanism of action of MT in the intervention of mental illness, cancer patients and analgesia should be further explored. Qualitative and quantitative integrated research ideas should be used to collect data such as imaging, vital signs and subjective feeling scales, so as to further optimize evidence-based research and improve the quality of research.

### 4.2. Practical significance of the research

This study conveys complicated facts in the field of MT and anxiety research through the data summarize and visualization. It is significant for hospitals, students, and institutions and researchers working in the fields of MT, anxiety, and sleep. Firstly, this study summarized the significant cooperation forces, classic and key literature and research hot topics in this field, as well as the research progress and emerging research hot topics in the field of MT and anxiety, which serves as a significant source of information for scientific research on mental illness in hospitals and universities. Secondly, this study began by summarizing the current state of the research on MT and anxiety and concludes that there are worthwhile future research directions that are still worth investigating in the clinical practice of MT and anxiety. Some of these directions include paying attention to mental health, utilizing rehabilitation nursing technology, and creating clinical evidence-based standard systems. This study has the potential to increase public understanding of MT and anxiety as well as mental health awareness, all of which are crucial for lowering the prevalence of mental diseases.

### 4.3. Strengths and restrictions

Based on our current knowledge, this is the first publication that employs bibliometric and visual analysis methods to more vividly examine trends and patterns in the field of MT and anxiety over a span of thirty years. The 1147 items in this study are focused on countries, institutions, journals, references, authors, and keywords to describe the macro analysis of the field, comprehend the current state of MT and anxiety research, demonstrate the background of this field’s research, and also contain burst references and burst keywords to help discover research hot topics and future trends in this field.

Nonetheless, this study does have certain restrictions. Firstly, we only took into account relevant literatures on the English database WoSCC owing to software and technical limitations, and we did not take into account other databases. Secondly, this study selected English as the inclusion criterion and excluded non-English articles, which could have influenced the published literature. Thirdly, the accuracy and scientific rigor of publications cannot be fully taken into account by bibliometrics, the timing of publications may have an impact on citation indicators, and new published studies may be underestimated; Finally, since this study is a macro analysis, there are some important detail parts that may not be included. But, we believe that the basis of our work can provide some effectively references to the global research in field of MT and anxiety.

## 5. Conclusion

The focus of this bibliometric study is to provide the most recent discoveries and potential future research directions in the field of MT and anxiety by conducting a visual analysis of the detailed information from the global literature in the field over the past 30 years. The number of studies on MT and anxiety showed a significant year by year increase in 2009, indicating that this research field has good potential for growth. The important authors in MT and anxiety field are Nilsson U, Bradt J, and Kuhlmann AYR and so on. the United States, China, Turkey, England, and Australia are the core research country, and the University of London is an important research institution, *The Journal of Music Therapy* is a representative core journal in the field. The main research direction is cancer anxiety, surgery and anxiety and pain in recent years. Besides, there are also scattered studies on quality of life, dementia, children, and nursing, which are worthy of further exploration and research in the whole social environment. The collaboration and communication among countries, academic institutions, and researchers may be enhanced as a result of this literature review. Additionally, it offers a helpful and valuable foundation for better comprehending research priorities, hotspots, and current developments in the field of MT and anxiety.

## Acknowledgments

The open source use of the advanced software developed by C.M.Chen, who developed the free software CiteSpace, Van Eck and Walterman of VOSviewer, and Vladamir Batagelj of Pajek is gratefully acknowledged.

## Author contributions

**Conceptualization:** Jin Yu.

**Data curation:** Tingting Lun, Li Li, Meng Xiang.

**Formal analysis:** Tingting Lun, Jin Yu.

**Funding acquisition:** Jin Yu.

**Investigation:** Yuecai Chen.

**Methodology:** Jin Yu, Meng Xiang.

**Project administration:** Jin Yu.

**Resources:** Jingcai Liu.

**Software:** Tingting Lun, Yuecai Chen, Li Li.

**Supervision:** Li Li.

**Visualization:** Yuecai Chen, Jingcai Liu.

**Writing – original draft:** Tingting Lun.

**Writing – review & editing:** Yuecai Chen, Jingcai Liu, Li Li, Meng Xiang.
